# 4121 The beneficial, anti-fibrotic effects of chemokine receptor 2 and 5 antagonists on fat-exposed mouse primary hepatic stellate cells (pHSCs)

**DOI:** 10.1017/cts.2020.317

**Published:** 2020-07-29

**Authors:** Annie J. Kruger, Kruger Bergman, Martha Gay, Hong Cao, Robin Tucker, Narayan Shivapurkar, Jill P. Smith

**Affiliations:** 1 Georgetown - Howard Universities; 2 St. Louis University School of Medicine; 3 Georgetown University

## Abstract

OBJECTIVES/GOALS: Non-alcoholic steatohepatitis (NASH) is a leading cause of cirrhosis in the world for which no anti-fibrotic therapies exist. We hypothesized that BMS-22 and maraviroc (MVC), chemokine receptor 2 (CCR2) and 5 (CCR5) antagonists, respectively, would diminish the fibrogenic activity of "fat-exposed" murine pHSCs. METHODS/STUDY POPULATION: pHSCs were isolated from livers of 6 week old male mice following 4 weeks on a NASH-inducing choline-deficient high fat diet (CDAHFD, “fat-exposed”) or standard diet (SD) and passaged *in vitro*. Early passage (6-12) pHSCs were plate-adhered and TGF-b-treated (10ng/mL) to maximally activate their pro-fibrogenic genes, *collagen 1α1* (*Col1A1), tissue inhibitor of metalloproteinase 1* (*TIMP1)*, or *α-smooth muscle actin (ACTA2*). CDAHFD and SD pHSCs were then treated for 48 hours with increasing doses of BMS-22 or MVC (range: 0.3-120ng/mL) to determine (1) the degree of attenuation of the pro-fibrogenic response as measured by qPCR of fibrogenic genes (*Col1A1, TIMP1,ACTA2);* (2) enhancement of a fibrolytic response as measured by qPCR of *matrix metalloproteinases (MMP*) *2, 9* and *13* genes; and (3) pHSC migration using the scratch assay. Cell viability and CCR2 and CCR5 gene expression in response to escalating doses of antagonists were also measured. RESULTS/ANTICIPATED RESULTS: Plate- and TGF-b activated CDAHFD pHSCs had a 2-fold greater, dose-dependent attenuation of their pro-fibrogenic activity in response to BMS-CCR2-22 and MVC, when compared with plate- and TGF-b activated SD pHSCs, as measured by reductions in collagen 1α1 (*Col1A1*) and α-smooth muscle actin (*ACTA2*) gene expression. *TIMP1* gene expression was unaffected by drug treatment for 48 hours. Cell viability was not affected up to doses of 30ng/mL of each drug. pHSCs also demonstrated a dose-dependent increase in *CCR2, CCR5* and *MMP-9* gene expression in response to surface receptor antagonism. Migration assays comparing CDAHFD and SD pHSCs in response to escalating doses of MVC and BMS-22 are ongoing and expected to demonstrate a significantly decreased migratory capacity of CDAHFD pHSCs than SD pHSCs in response to therapy, reflecting the increased susceptibility of the “fat-exposed” pHSCs to anti-fibrotic therapy than normal pHSCs. DISCUSSION/SIGNIFICANCE OF IMPACT: Anti-fibrotic drugs that dampen pro-fibrogenic activities of “fat-exposed” pHSCs are urgently needed. CCR2 and CCR5 antagonists, BMS-22 and MVC, respectively, can selectively dampen the pro-fibrogenic response of fat-exposed pHSCs, and must be considered for future trials in human NASH. CONFLICT OF INTEREST DESCRIPTION: Dr. Jill Smith has a patent licensing agreement with Immune Therapeutics, Inc.

